# Roadmap for understanding mechanisms on how Epstein–Barr virus triggers multiple sclerosis and for translating these discoveries in clinical trials

**DOI:** 10.1002/cti2.1438

**Published:** 2023-02-16

**Authors:** Tobias V Lanz, William H Robinson, Peggy P Ho, Lawrence Steinman

**Affiliations:** ^1^ Stanford University Stanford CA USA

**Keywords:** Epstein–Barr virus, GlialCAM, molecular mimicry, multiple sclerosis

## Abstract

Here, we offer a roadmap for what might be studied next in understanding how EBV triggers MS. We focus on two areas: The first area concerns the *molecular mechanisms* underlying how clonal antibody in the CSF emanates in widespread molecular mimicry to key antigens in the nervous system including GlialCAM, a protein associated with chloride channels. A second and equally high priority in the roadmap concerns various *therapeutic approaches* that are related to blocking the mechanisms whereby EBV triggers MS. Therapies deserving of attention include clinical trials with antivirals and the development of ‘inverse’ vaccines based on nucleic acid technologies to control or to eradicate the consequences of EBV infection. High enthusiasm is given to continuation of ongoing clinical trials of cellular adoptive therapy to attack EBV‐infected cells. Clinical trials of vaccines to EBV are another area deserving attention. These suggested topics involving research on mechanism, and the design, implementation and performance of well‐designed trials are not intended to be an exhaustive list. We have splendid tools available to our community of medical scientists to tackle how EBV triggers MS and then to perhaps change the world with new therapies to potentially eradicate MS, as we have done with nearly complete success for poliomyelitis.

## Graphical abstract

We provide a roadmap for future investigations on how Epstein–Barr virus triggers multiple sclerosis and how this may be translated in the clinic.

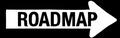

## Introduction

Having lectured, often virtually, all over planet Earth about the breakthroughs in 2022 regarding Epstein–Barr virus (EBV) and multiple sclerosis (MS), we offer some personal ideas regarding key questions to explore in the years ahead. These suggested topics involve research on mechanism and performance of well‐designed trials. Research on the mechanistic basis for how EBV triggers MS may also illuminate parallel mechanisms in other diseases including systemic lupus erythematosus, rheumatoid arthritis, Sjogren's syndrome, Long COVID and even myalgic encephalomyelitis/chronic fatigue syndrome.

## Understanding mechanism about how EBV triggers MS

### Multiple molecular mimics in a short stretch of EBNA‐1

Work led by Professor Alberto Ascherio for more than a decade at the Harvard School of Public Health pointed to EBV as a critical trigger of MS. The publication in Science from Bjornevik and colleagues^1^ published in January 2022 had an extraordinary hidden gem, a ‘diamond in the rough’, in table S1 in their seminal publication. Antibody levels to various EBV proteins in MS patients showed the strongest signal compared with controls when the region of the peptide of EBNA‐1 between amino acids 365 and 420 was interrogated.^1^ This region includes a stretch of EBNA‐1 shared with a molecule called GlialCAM.^2^ Another study^3^ showed serum antibodies from MS patients are cross‐reactive between amino acids 411–440 of EBNA‐1 and the human chloride channel protein, anoctamin 2 (ANO2), which is associated with electrical conduction in axons.^3^ MS serum antibodies targeting EBNA‐1 residues 411–426 that cross‐react with myelin basic protein have also been identified.^4^

Clonally expanded antibodies isolated from the cerebrospinal fluid of MS patients target EBNA‐1 residues 386–405 that cross‐react with the CNS cell adhesion molecule GlialCAM and are associated with CD4 and CD8 T cell responses targeting GlialCAM. These T cells produce the cytotoxic protein called granzyme and thus might kill cells bearing this antigen. This molecular mimicry was described in a paper in Nature, published shortly after Ascherio's study appeared in Science.^1^, ^2^, ^5^ The clonal antibody expressed in the B cell lineage may act as a potent binder of antigens associated with MS like GlialCAM. Antigen presentation by B cells may be a critical step in the pathogenesis of MS. Antigen presentation via B cells to CD4 and CD8 T cells may allow these cytotoxic cells to become activated. We detected CD8 T cells recognising GlialCAM and producing granzyme, which would allow them to kill oligodendrocytes and even astrocyte end‐feet bearing this target.^2^ These CD8 T cells recognised GlialCAM epitopes in both the intracellular and extracellular domains.^2^ One MS patient showed extraordinarily high counts of IFNγ^+^ granzyme B^+^CD8^+^ T cells after stimulation with EBNA1 and the intracellular domain of GlialCAM.^2^

It is intriguing that three contiguous regions of mimicry have been reported in a small region of the EBNA‐1 protein. This hotspot for molecular mimicry may arise through immune surveillance in a process called epitope spreading.^6^ In epitope spreading, immunity to molecules in a common anatomic location may ensue, once tissue‐specific autoimmune disease commences. Two molecules related to chloride channels, GlialCAM and anoctamin 2,^7^, ^8^ and the major protein in the myelin sheath, myelin basic protein,^4^ may all come under attack because of their anatomic proximity to one another in the paranodal regions of an axon and in the nearby myelin sheath. If all three molecules have mimicry with EBNA‐1, then the molecular mimicry ‘hotspot’ may be a result of this anatomic confluence of pathophysiologic events targeting three molecules that are in the same ‘immunological region of attack’.^6^ Using a military metaphor, the diameter of the bomb encompasses not only its point of contact with the target but also triggers collateral damage to the surround.

It is important to note that at this point studies in the blood indicate that only about 15% of MS patients have detectable antibody to anoctamin 2,^3^ and about 25% of MS patients have antibody in blood to GlialCAM.^2^ Longitudinal studies on the persistence of these antibodies in blood and in CSF are underway. It will be important to learn whether the antibodies are ephemeral or persistent, whether a ‘hit and run’ encounter might be sufficient to ignite disease, and whether the persistence of these antibodies is important for disease progression. Within the CSF are clonal antibodies to other infectious viruses like cytomegalovirus and varicella zoster, which may be associated perhaps, with progression and even with relapses.

Mutations in GlialCAM are involved in a rare paediatric neurologic disease called megalencephalic leukodystrophy with subcortical cysts. This disease is caused by mutations either in a protein called MLC‐1 or in GlialCAM.^7^ The GlialCAM gene encodes a protein that acts as a chaperone between the MLC‐1 protein and the ClC‐2 chloride channel.^8^ It is fascinating that anoctamin‐2^3^ is itself a chloride channel, whereas GlialCAM acts as a chaperone for the proper clustering at the cell–cell interface of the ClC‐2 chloride channel.

Electrophysiologists and biochemists could help unravel the curious mechanistic role of chloride channels in two of the molecular mimics appearing so close together within EBNA‐1.

Another curiosity is the expression of GlialCAM primarily in oligodendrocytes in the central nervous system, but also at very low levels in the liver. The gene was first described in hepatocellular carcinoma and was named HepaCAM. Its expression in normal liver is very low.^9^ Antibodies to GlialCAM are present in the cerebrospinal fluid (CSF) of MS patients and at any one point in time appear in the serum of about 25 to 30% of MS patients. The liver is spared in MS, so it will be interesting to understand why the nervous system is attacked in MS, but the liver is spared. It may simply be because of the fact that its expression is so very low in normal hepatic tissue. Of course, there are other examples of proteins like aquaporin 4 (AQP4) where nervous system disease is triggered when AQP4 is attacked by antibodies in neuromyelitis optica (NMO),^10^ while the kidney, where AQP4 is also present, is spared in NMO.^11^ There are many other examples of widespread antigens that are attacked in organ‐specific autoimmunity, sparing most of the locations where the widespread antigen is expressed.

### GlialCAM and paranodal proteins share similar structures

GlialCAM is a member of the immunoglobulin supergene family, one of the four major types of adhesion molecules. The three other types of cellular adhesion molecules are integrins, cadherins and selectins. The paranodal proteins, contactin, contactin‐associated protein and neurofascin are members of this family and share significant homology with GlialCAM. Neurofascin has a 29 per cent identity with GlialCAM, while contactin‐1 has a 24 per cent identity with GlialCAM. Explorations of cross‐reactive antibodies to EBNA‐1 with these paranodal proteins in inflammatory neuropathy are indicated. Infections with EBV account for about 10% of GBS cases.^12^ Antibodies to paranodal proteins are found in MS and in both Guillain–Barre syndrome and chronic inflammatory polyneuropathy.^13^

### MEF2b like GlialCAM binds to clonal antibody in the CSF MS: This may Be critical for the development of lytic cells in the brain in MS

In figure 3 in the paper by Lanz, Robinson and colleagues,^2^ we noted strong binding of the monoclonal antibody, which also bound to GlialCAM, to a transcription factor known as MEF2b. MEF2b has some homologies with GlialCam including some proline and arginine‐rich regions. MEF2b binds to EBNA‐1.^14^ EBNA‐1 regulates interleukin 6 receptor (IL6R) and MEF2b and is responsible for the viability of EBV‐infected cells. EBNA‐1 and MEF2b play a role in suppressing the lytic cycle. One could imagine that an antibody binding MEF2b might block its propensity to suppress the lytic cycle of EBV, thereby activating the lytic phase of EBV. Blocking a suppressive molecule activates the pathway that the molecule, in this case MEF2b was suppressing. It is very interesting that this was the second most active ‘hit’, when we explored the human proteome with some of the clonal antibodies in the CSF. It is intriguing that the same monoclonal bound to GlialCAM and bound to MEF2b. These molecules contain the same binding motif PPRRPP, which likely explains the immunological ‘cross‐reaction’. Blocking PPRRPP binding with cleverly designed small molecules might have therapeutic potential.

There are other molecular mimics that share this motif including a molecular mimic between the U24 membrane protein for HHV‐6A and myelin basic protein^15^ and between GlialCAM and members of the poxvirus family.^16^ Such phenomena are sometimes referred to as the ‘dual virus’ hypothesis, which suggests that EBV might upregulate a second virus. Of interest, infection with SARS‐CoV2 may regulate EBV itself and thus contribute to the many similar disabilities including fatigue and ‘brain fog’ associated with Long COVID and MS.^17^

### Plasmablasts have high integrin alpha 4 (ITGA4) in blood and may be trapped when they lose this key homing molecule

α4 integrin (ITGA4) is a major therapeutic target in MS.^15^ We showed that levels of α4 integrin (ITGA4) were reduced on plasmablasts in the CSF compared with their level in blood.^2^ Integrin α4 is the critical homing molecule into the brain for T cells, B cells and plasmablasts in MS.^18^ Whether this loss of α4 integrin leads to the retention of plasmablasts in the CSF compartment ought to be studied. The role of α4 integrin has been actively studied in haematology regarding trafficking and retention in the bone marrow,^19^ but studies are begging to be performed in neuroinflammation regarding trafficking into and out from the brain compartment. A role for the glymphatics and meningeal lymphatic drainage in this process also deserves attention in relation to the loss of α4 4 integrin in the CSF.^20^

### Potential role for EBV‐mediated B cell transformation in MS

It is likely that mechanisms beyond molecular mimicry contribute to the role of EBV in MS. EBV establishes latent infection of B cells, and ‘latently infected’ B cells express multiple EBV genes and RNAs including LMP1, LMP2, EBNA1, EBNA2 and EBERs that can contribute to B cell activation. It is possible that EBV‐mediated transformation of autoreactive B cells could provide a shared mechanism underlying MS as well as other human autoimmune diseases.

## New therapeutic approaches deserving attention addressing EBV

### Antiviral trials and development of vaccines against EBV

Trials of antiviral therapeutics in MS seem particularly relevant. Antivirals that have activity against herpes viruses particularly EBV remain the most relevant to take forward. Valomaciclovir has shown efficacy in trials of herpes zoster^21^ and in a small trial on students with infectious mononucleosis.^22^, ^23^ An antiviral like valomaciclovir that penetrates the blood–brain barrier would be optimal, since it would address the CSF and meninges where there are ‘nests’ of B cells and plasmablasts that are producing anti‐GlialCAM antibodies. Techniques to address the lytic phase of disease over the preponderance of B cells that remain in the latent phase would be desirable. Lytic EBV virus appears mainly in more chronic lesions of MS.^24^ Therapeutic approaches that induce the lytic stage of EBV in B cells, followed by antiviral treatment, for example with valganciclovir are ongoing in non‐MS EBV‐mediated diseases (https://ashpublications.org/blood/article/138/Supplement%201/623/479908/Nanatinostat‐Nstat‐and‐Valganciclovir‐VGCV‐in). This strategy could be adopted for MS treatment, albeit it is unclear as to what effect a systemic reactivation of EBV would have on neuroinflammation.

Tenofovir, an antiviral medication used to treat HIV‐1 and chronic hepatitis B, was recently shown to be a potent inhibitor of EBV lytic DNA replication.^25^ Tenofovir was listed on clinicaltrials.gov as an add‐on to anti‐CD20 therapies. That trial was withdrawn, but trials are being proposed with Tenofovir and other antivirals in various locations on our planet.^26^ It will be important to see whether antiviral activity against both lytic and latent phases of EBV infection will be relevant.

### Continuation of ongoing clinical trials of cellular adoptive therapy attacking EBV‐infected cell

An ongoing trial in progressive MS continues with a premanufactured, unrelated donor (off‐the‐shelf, allogeneic) EBV‐targeted T cell immunotherapy comprised of partially HLA‐matched, *in vitro* expanded, cytotoxic T lymphocytes, specific for EBV protein antigens. Work is based on pioneering studies from Michael Pender and Rajiv Khanna,^27^, ^28^, ^29^ which have shown promise in early‐stage trials. In the initial trial performed by Khanna and Pender,^28^ ‘all 6 patients receiving T cells with strong EBV reactivity showed clinical improvement, whereas only 1 of the 4 patients receiving T cells with weak EBV reactivity showed improvement (*P* = 0.033, Fisher's exact test)’. There are two ongoing clinical trials with autologous and allogeneic *in vitro* expanded EBV‐specific CD8 T cells.

Small molecule inhibitors of EBNA‐1 binders have been designed and are worthy of consideration. As mentioned previously, design of molecules that block the PPRRPP motif might prove useful.^2^

### Immunisation to EBV with mRNA technologies as well as with conventional technologies

In January of 2022, Moderna announced the initiation of an mRNA vaccine to immunise against EBV. The Eclipse trial will test the experimental mRNA vaccine against 4 envelope glycoproteins in 18‐ to 30‐year‐old healthy adults.^30^ Interestingly, Moderna is developing mRNA vaccines that encode envelope glycoproteins and could prevent initial infections with EBV. However, while its efficacy in suppressing symptomatic infectious mononucleosis and other EBV‐related diseases seems achievable, it is unclear whether sterile protection will be possible.

By contrast, another mRNA vaccine under development encodes latent EBV antigens and could therefore generate an immune response against latent EBV‐infected B cells long after the initial infection. The second strategy seems highly relevant for the treatment of MS patients. Our studies that identified molecular mimicry regions could guide the development of these vaccines, as those regions ought to be avoided in the vaccines.

### Tolerisation to GlialCAM with nucleic acid ‘inverse vaccines’ and with conventional technologies

For the past 20 years, we have been pursuing antigen‐specific tolerance with an engineered DNA vaccine encoding myelin antigens, where inflammatory CpG hexanucleotides were deleted from the noncoding portion of the DNA plasmid. Initially, we tested such a vaccine where four major myelin proteins, myelin basic protein, proteolipid protein, myelin oligodendroglial glycoprotein and myelin‐associated glycoprotein were encoded in separate circular DNA plasmids designed to tolerise rather than immunise. We sought to suppress the animal model of MS, known as experimental autoimmune encephalomyelitis (EAE). Initial results were promising^31^ and showed that we could reduce the relapse rate for EAE in mice immunised with myelin. In these preclinical studies, we also observed a reduction in the levels of antibody to these same myelin proteins. This approach was taken into the clinic in phase 1^32^ and phase 2 clinical trials in 300 patients with relapsing–remitting MS.^33^

In the phase 2 trial on 267 patients with relapsing MS, monthly treatments for 44 weeks with 0.5 mg of a plasmid, designed to tolerise, which encoded myelin basic protein, nearly attained the primary end point for reduction of the rate of new enhancing magnetic resonance imaging (MRI) lesions (*P* < 0.07). The trial achieved several secondary end points. A prespecified secondary endpoint showed reduction in the rate of enhancing MRI lesions from Weeks 8 to 48 (*P <* 0.05).^33^ There were 61% fewer lesions on the Weeks 8–48 MRIs of patients with 0.5 mg BHT‐3009 than those on the placebo (*P <* 0.05). More recently, we reported that a tolerising DNA plasmid encoding GlialCAM could suppress EAE.^34^

Meanwhile, investigators at BioNTech and the University of Mainz redesigned the mRNA vaccine technology that was spectacularly successful in making an effective vaccine to SARS‐CoV‐2. The technology was ‘repurposed’ to develop a tolerising vaccine.^35^ The investigators showed that ‘delivery of nanoparticle‐formulated 1 methylpseudouridine‐modified messenger RNA (m1Y mRNA) coding for disease‐related autoantigens results in antigen presentation on splenic CD11c^+^ antigen‐ presenting cells in the absence of costimulatory signals’. The tolerising mRNA vaccine suppressed EAE in various models. Thus, nucleic acid‐based ‘inverse vaccines’ may be valuable for tolerising the immune system to GlialCAM and to other relevant molecules in the pathogenesis of MS.

It might be reasonable to wonder how studies on EAE might help us understand virus‐induced model of autoimmune disease. Recent bioinformatic studies show that the PPRRPP motif is found in other viruses, not merely EBV. In fact, pox viruses and HHV‐6 share this motif. Immunisation with this peptide motif in mice with ongoing experimental autoimmune encephalomyelitis (EAE) can worsen paralysis.^2^ This 90‐year‐old animal model, known as EAE, might be a convenient system to attempt tolerisation to this motif that is shared between many different viruses and molecules in the nervous system. Of historical interest, the EAE model was originally devised by Thomas Rivers to test why smallpox virus might trigger disseminated encephalomyelitis. This virus carries the GlialCAM motif associated with neuroinflammation.^16^

There are other approaches to tolerising the immune system under testing in the clinic that involve approaches other than DNA and mRNA vaccines. These approaches include the use of nanoparticles containing autoantigenic proteins or fragments of these proteins, as well as approaches where autoantigens are conjugated chemically to red blood cells.^36^, ^37^

### Conclusion

This review summarises some key questions regarding the mechanism of how EBV triggers MS, and provides some thoughts on how we might translate current knowledge into clinical trials for MS. It has been an exciting year since the publications on EBV and MS in early 2022.

## Conflict of Interest

Lawrence Steinman has issued patents and an ongoing application on DNA vaccines for tolerance to myelin antigens, including GlialCAM. Lawrence Steinman also has patent filings regarding antivirals for the treatment of MS. Lawrence Steinman is the principal investigator at Stanford University in the ATA188 trial with a cell‐based therapy for progressive MS. William Robinson and Tobias Lanz have patent filings relevant to these studies.

## Author Contributions

**Tobias Volker Lanz:** Conceptualization; data curation; investigation; writing – original draft; writing – review and editing. **William H Robinson:** Formal analysis; funding acquisition; investigation; writing – original draft; writing – review and editing. **Peggy P Ho:** Investigation; writing – original draft; writing – review and editing.
